# Supplementary material for: An intrinsically disordered nascent protein interacts with specific regions of the ribosomal surface near the exit tunnel
Source: Commun Biol. 2021 Oct 29;4:1236. doi: 10.1038/s42003-021-02752-4 (PMC8556260; doi:10.1038/s42003-021-02752-4)
Supplement: Supplementary file 2 — Supplementary Information [file 42003_2021_2752_MOESM2_ESM.pdf]

SUPPLEMENTARY INFORMATION

An intrinsically disordered nascent protein interacts with specific regions of the ribosomal surface near the exit tunnel

Valeria Guzman-Luna[1], Andrew M. Fuchs[1], Anna J. Allen[1], Alexios Staikos[1] and Silvia Cavagnero[1]

[1] Department of Chemistry, University of Wisconsin-Madison, 1101 University Ave., Madison, Wisconsin, 53706, USA

Correspondence should be addressed to S.C. (E-mail: cavagnero@chem.wisc.edu, Phone: 608-262-5430)

Supplementary Tables

Table S1. Summary of ribosomal proteins known to interact with nascent chains bearing translational stalling or signal sequences.

RNC	Technique	Interacting ribosomal protein	Interaction-site location relative to the ribosomal tunnel (inside / outside)
TnaC[1]	photo-crosslinking	L22 and L4	inside *(E. coli)*
TnaC[2]	cryo-EM	L22	inside *(E. coli)*
Leader peptidase (Lep)[3]	photo-crosslinking	L4, L22 and L23	inside *(E. coli)*
SecM[4]	cryo-EM	L22 and L23	inside *(E. coli)*
SecM[5]	mutagenesis	L22	inside *(E. coli)*
Two regulatory peptides[6]	cryo-EM	L4 and L17	inside (eukaryotic ribosome)
Signal anchor of Dap2 protein[7]	photo-crosslinking	Rpl4, Rpl17, and Rpl39	inside (eukaryotic ribosome)
Transmembrane segment of 111p membrane protein[8]	photo-crosslinking	N.D.	outside *(E. coli)*
Signal anchor of FtsQ protein[9]	photo-crosslinking	L23 and L29	L23 and L29: in and out
pOmt[10]	photo-crosslinking	L23, L24	outside *(E. coli)*

Signal peptide (EspP)[11]	photo-crosslinking	L23 and L24	outside *(E. coli)*
SecM[12]	cryo-EM	L23	outside (E. coli)

Table S2. Molecular weight of ribosomal proteins belonging to the large 50S (RL) and small 30S (RS) subunits of the *E. coli* ribosome (strain K12). [13]

30S Ribosome subunit			50S Ribosome subunit		
**Ribosomal protein (RS)**	**Uniprot ID**	**MW (kDa)**	**Ribosomal protein (RL)**	**Uniprot ID**	**(MW kDa)**
21	P68679	8.4	36	P0A7Q6	4.4
18	P0A7T7	8.9	34	P0A7P5	5.4
16	P0A7T3	9.2	33	P0A7N9	6.2
17	P0AG63	9.6	32	P0A7N4	6.3
20	P0A7U7	9.6	30	P0AG53	6.4
15	P0ADZ4	10.1	35	P0A7Q1	7.2
19	P0A7U3	10.3	29	P0A7M6	7.3
14	P0AG61	11.4	31	P0A7M9	7.9
10	P0A7R5	11.7	28	P0A7M2	8.9
13	P0A7S9	13.0	27	P0A7L8	9.0
12	P0A7S3	13.6	25	P68919	10.7
11	P0A7R9	13.7	23	P0ADZ0	11.2
8	P0A7W7	14.0	24	P60624	11.2
9	P0A7X3	14.7	21	P0AG48	11.6
6	P02358	15.2	22	P61175	12.2
6	P02358	15.7	7	P0A7K2	12.2
5	P0A7W1	17.5	18	P0C018	12.8
7	P02359	19.9	19	P0A7K6	13.0
4	P0A7V8	23.3	20	P0A7L3	13.4
3	P0A7V3	25.9	14	P0ADY3	13.5
2	P0A7V0	26.6	17	P0AG44	14.4
1	P0AG67	61.2	11	P0A7J7	14.7
			15	P02413	15.0
			16	P0ADY9	15.3
			9	P0A7R1	15.8
			13	P0AA11	16.0
			10	P0A7J3	17.6
			6	P0AG55	18.8
			5	P62399	20.2
			4	P60723	22.1
			3	P60438	22.2
			1	P0A7L0	24.6
			2	P60422	29.7

Supplementary Figures

Fig. S1

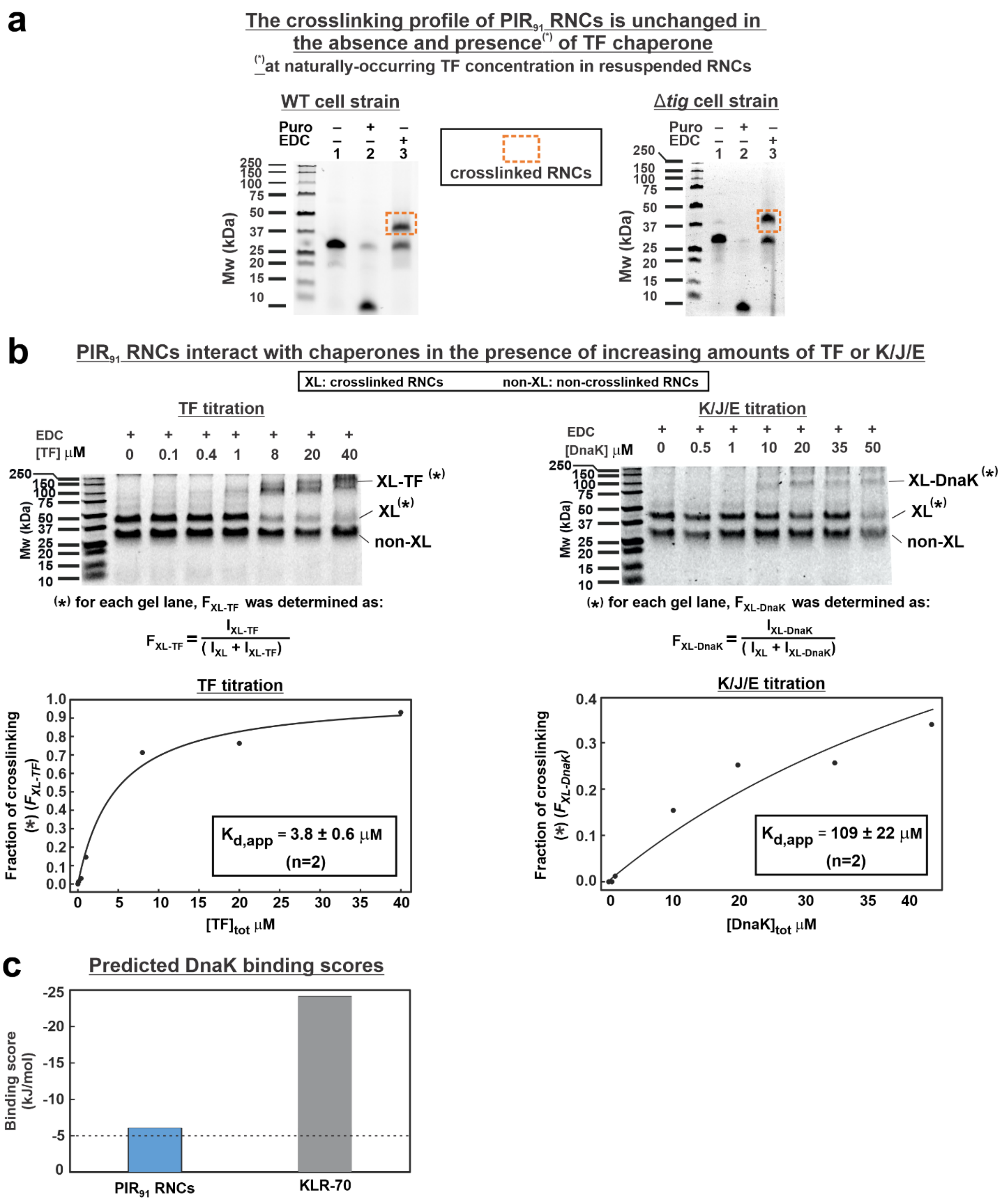

Fig. S1. Crosslinking profile of PIR_{91} RNCs in the absence and presence of molecular chaperones. (a), Crosslinking profile of PIR_{91} RNCs produced in wild-type *E. coli* S30 extract (left), and crosslinking profile of PIR_{91} RNCs produced in *Δ-tig E. coli* S30 extract, which is devoid of the trigger-factor (TF) chaperone gene (right). **(b),** Representative experiments showing the crosslinking profile of PIR RNCs in the presence of increasing concentrations of *E. coli* TF or Hsp70 (DnaK, denoted here as K), DnaJ (J), and GrpE (E) (K/J/E) chaperones. The TF assays were carried out upon resuspension of pelleted PIR RNCs (generated via cell-free transcription-translation) in standard resuspension buffer (10 mM tris-HCl, 10 mM $Mg(OAc)_2$, 60 mM NH_4Cl, 0.5 mM EDTA and 1.0 mM DTT, pH 7.0). Crosslinking assays on ADP-DnaK were carried out at K/J/E 5:1:2 molar ratios in the presence of 5 mM ADP, 50 mM KCl, and resuspension buffer containing 5 mM Mg^{+2}. RNCs were first incubated in the presence of chaperones (10 min, 4 ºC) and then exposed to the EDC crosslinking agent. The plots include the apparent dissociation constant K_d ($K_{d,app}$ = avg ± S.E., for n = 2) derived from the fitting of individual titration data to Equation 6 (see Methods). Note that the maximum chaperone concentrations employed in the titrations are similar to the known cell-relevant concentrations of TF (40-50 μM)[14,15] and DnaK (50 μM)[16]. The resuspended RNC concentrations employed here (40 nM) are much lower than total *E. coli* RNC concentration (41.5 μM)[17]. However, given that the *in vivo* RNC concentration of individual expressed proteins is much lower than 41.5 μM and that the free TF and DnaK bioavailable concentrations for the binding to RNCs carrying specific nascent proteins is likely also much lower than 40-50 μM, it is hard to tell whether interactions of PIR_{91} RNCs with TF and DnaK actually happen in live *E. coli* cells. In any case, the $K_{d,app}$ values determined here provide a useful experimental assessment of the low affinity of PIR_{91} RNCs for the TF and DnaK chaperones. **(c),** Predicted binding score for the interaction of DnaK chaperone with PIR RNCs (focusing on the region not buried in ribosomal tunnel core, which exhibits only one DnaK binding site), according to Rudiger *et al*.[18] For comparison purposes, the binding score for the DnaK inhibitor KLR-70[19] is also shown. Binding scores lower than −5 kJ/mol denote the presence of a DnaK binding site.[18] Consistent with the experimental results of panel b, PIR is predicted to interact only weakly with DnaK.

Fig. S2

a

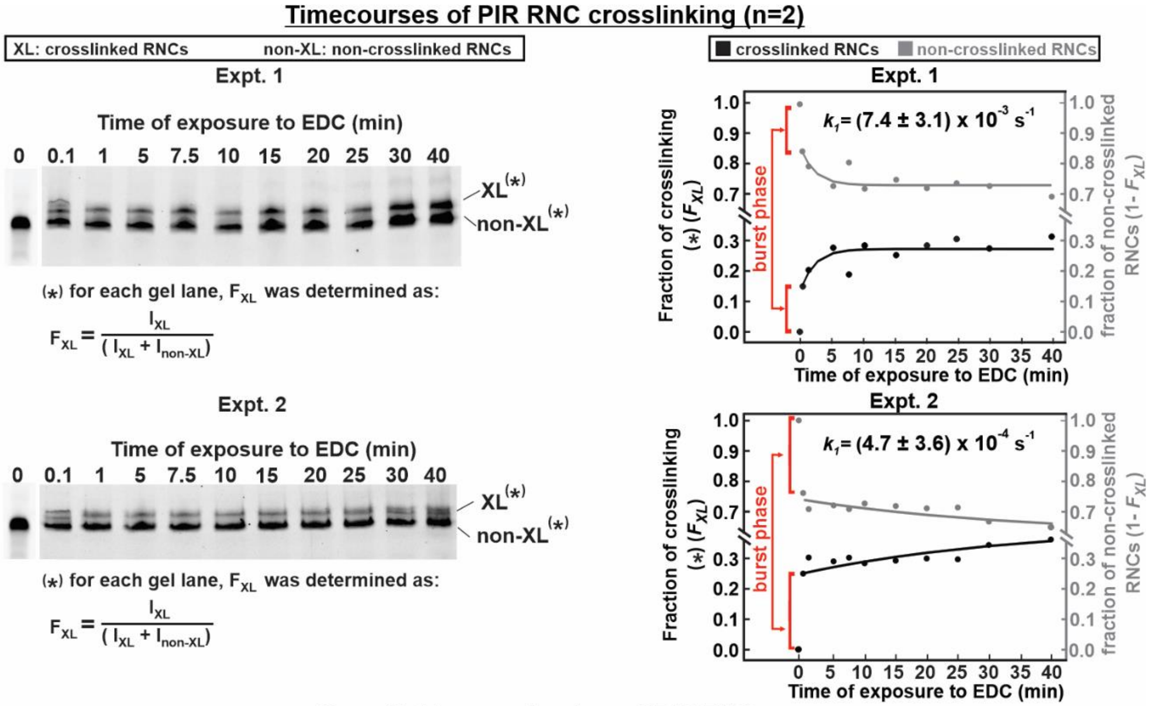

Timecourses of PIR RNC crosslinking (n=2)
XL: crosslinked RNCs
non-XL: non-crosslinked RNCs
Expt. 1
Time of exposure to EDC (min)
0 0.1 1 5 7.5 10 15 20 25 30 40
XL(*)
non-XL(*)
(*) for each gel lane, F_XL was determined as:
F_XL = I_XL / (I_XL + I_non-XL)
Expt. 2
Time of exposure to EDC (min)
0 0.1 1 5 7.5 10 15 20 25 30 40
XL(*)
non-XL(*)
(*) for each gel lane, F_XL was determined as:
F_XL = I_XL / (I_XL + I_non-XL)
crosslinked RNCs
non-crosslinked RNCs
Expt. 1
k1 = (7.4 ± 3.1) x 10^-3 s^-1
burst phase
Fraction of crosslinking (*) (F_XL)
fraction of non-crosslinked RNCs (1- F_XL)
Time of exposure to EDC (min)
Expt. 2
k1 = (4.7 ± 3.6) x 10^-4 s^-1
burst phase
Fraction of crosslinking (*) (F_XL)
fraction of non-crosslinked RNCs (1- F_XL)
Time of exposure to EDC (min)

b

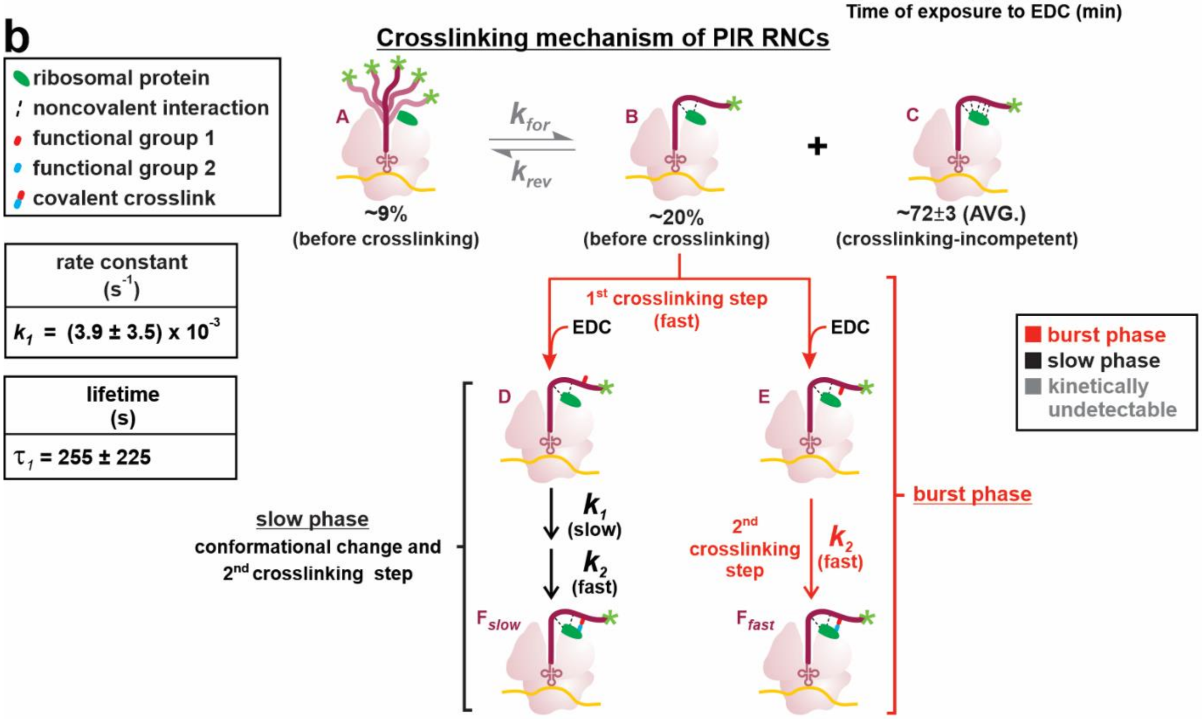

Crosslinking mechanism of PIR RNCs
ribosomal protein
noncovalent interaction
functional group 1
functional group 2
covalent crosslink
rate constant (s^-1)
k1 = (3.9 ± 3.5) x 10^-3
lifetime (s)
τ1 = 255 ± 225
A
k_for
k_rev
B
+
C
~9% (before crosslinking)
~20% (before crosslinking)
~72±3 (AVG.) (crosslinking-incompetent)
1st crosslinking step (fast)
EDC
EDC
D
E
burst phase
slow phase
kinetically undetectable
slow phase
conformational change and 2nd crosslinking step
k1 (slow)
k2 (fast)
2nd crosslinking step
k2 (fast)
burst phase
F_slow
F_fast

c

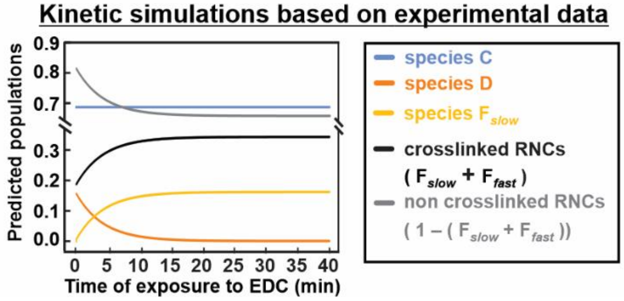

Kinetic simulations based on experimental data
Predicted populations
Time of exposure to EDC (min)
species C
species D
species F_slow
crosslinked RNCs (F_slow + F_fast)
non crosslinked RNCs (1 – (F_slow + F_fast))

Fig. S2 (cont.)

d

Residuals of kinetic data fits

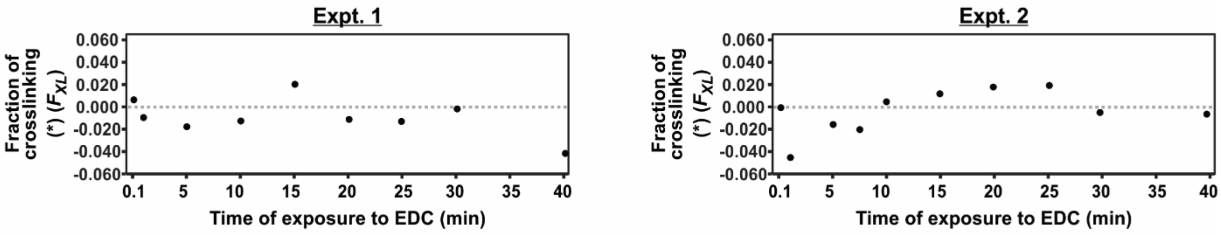

e

Reduced chi-square (X^2_R) values of kinetic data fits

Experiment	X^2_R
1	1.84
2	0.75

Fig. S2. Timecourse of crosslinking kinetics. (a), Raw data illustrating the timecourse of PIR RNCs crosslinking to ribosomal proteins for two independent experiments (left), and plots illustrating the corresponding time dependence of the crosslinking fraction (right). The plots include a rate constant (k_1; fit value ± fit error) derived from the fitting of individual crosslinking timecourse data to the slow phase of the crosslinking model of Fig. S2b. **(b),** Proposed mechanism of PIR RNC crosslinking. Arrows in gray, orange and black denote non-explicitly-modelled, burst-phase, and explicitly-modeled steps, respectively. Data fitting was carried according to a double-exponential function that includes a slow conformational step described by k_1, followed by a faster step accounting for the second crosslinking stage (k_2, fixed to 1×10^9 s^{-1}). **(c),** Kinetic simulations employing the averaged rate constant derived from the fits ($k_1 = 3.9 \pm 3.5 \times 10^{-3}$) and the known burst-phase amplitude. **(d),** Residuals of kinetic data fits according to the model in panel b (black steps only). **(e),** Reduced chi-square (χ^2_R) values of kinetic data fits, assessed as described in the Methods.

Fig. S3

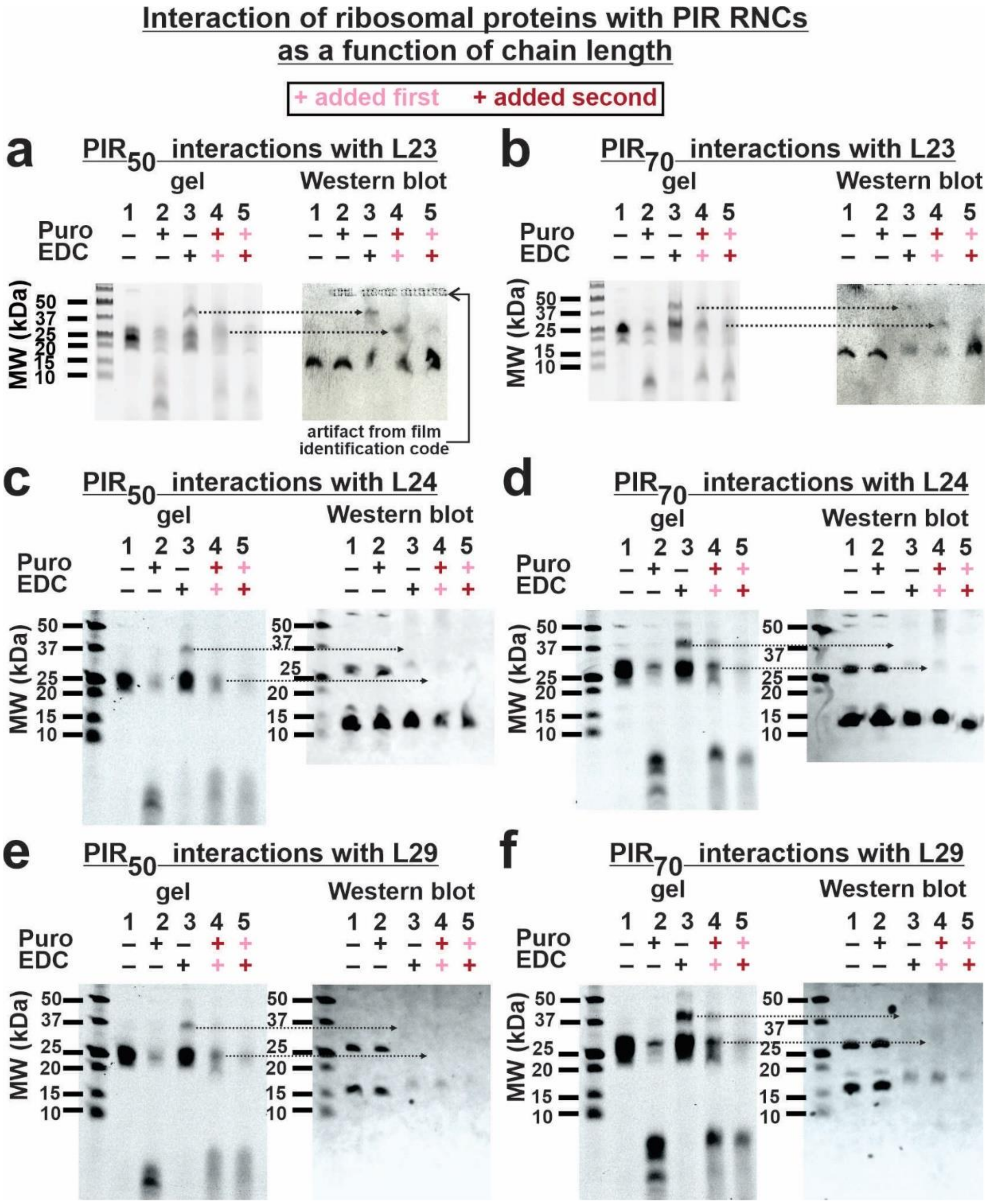

Fig. S3. SDS-PAGE and corresponding Western-blot data supporting the identification of ribosomal proteins crosslinked to nascent PIR_{50} and PIR_{70}. (**a, c, e**) SDS-PAGE of fluorescently-labeled PIR RNCs (left) of PIR_{50} and (**b, d, f**) SDS-PAGE of fluorescently-labeled PIR RNCs (left) of PIR_{70} and corresponding Western blotting against ribosomal proteins L23 (**a** and **b**), L24 (**c** and **d**) and L29 (**e** and **f**). Representative data are displayed, out of n = 2.

Fig. S4

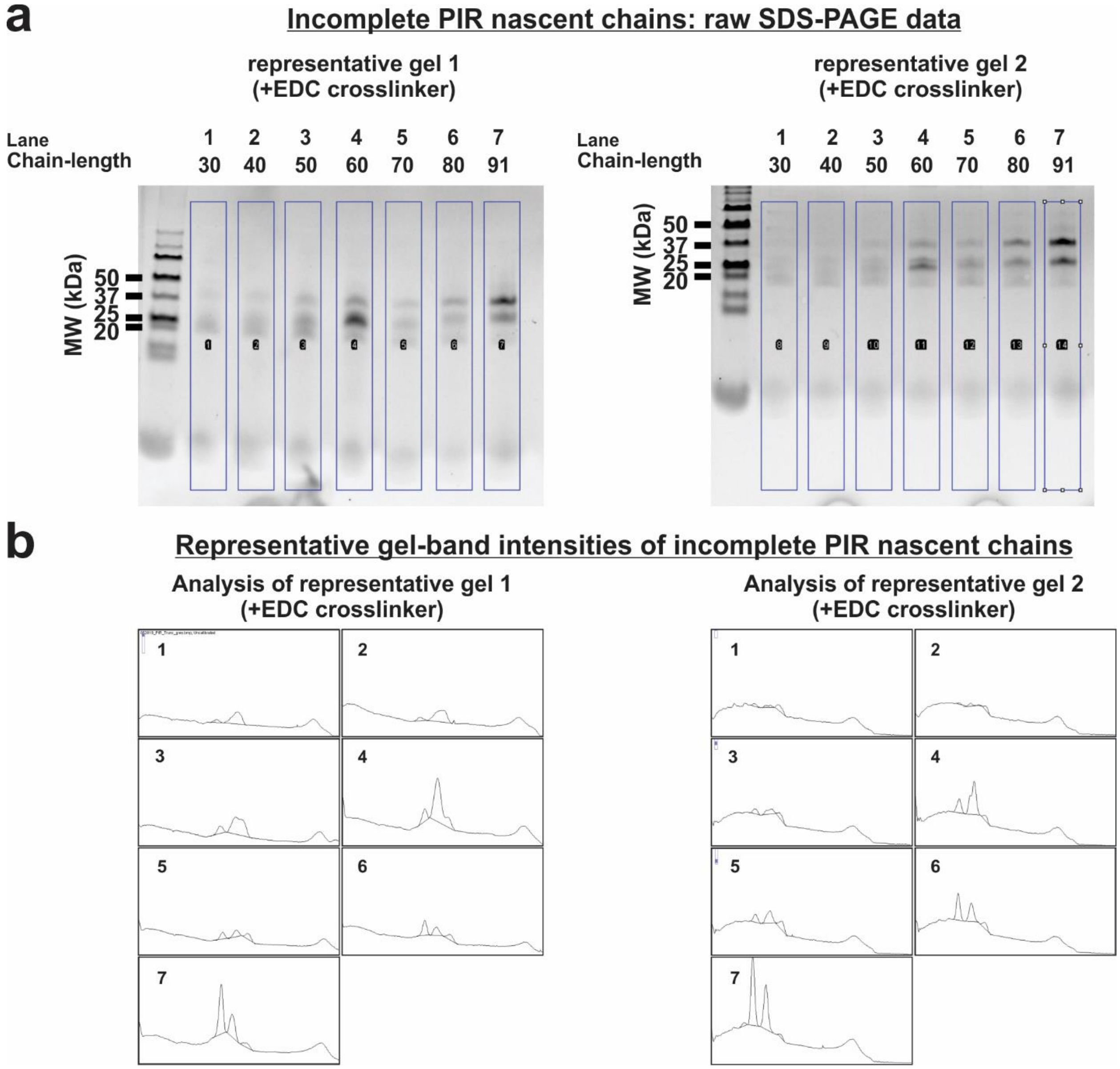

Fig. S4. Quantitative evaluation of crosslinked fraction of PIR nascent chains (RNCs) of increasing length. (a), Representative SDS-PAGE of crosslinked PIR nascent chains, from PIR_{30} to PIR_{91}. **(b),** Quantitative analysis of intensity of crosslinked and non-crosslinked RNC bands via the ImageJ software package. The individual peaks for crosslinked and non-crosslinked band were defined using the line tool. Intensities were then quantified. The baseline-related background of each peak was manually subtracted via the rolling-ball method according to recommended ImageJ-software guidelines.

Fig. S5

a **p values comparing PIR incomplete chains: fractions of crosslinked RNCs (Figure 5b, lefthandside plot)**

	PIR 30	PIR 40	PIR 50	PIR 60	PIR 70	PIR 80	PIR 91
PIR 30	-----						
PIR 40	0.61	-----					
PIR 50	0.01	0.05	-----				
PIR 60	0.00	0.01	0.35	-----			
PIR 70	0.00	0.00	0.01	0.10	-----		
PIR 80	0.00	0.03	0.00	0.00	0.00	-----	
PIR 91	0.00	0.00	0.00	0.00	0.00	0.00	-----

same / different

b **p values comparing PIR incomplete chains: normalized fractions of crosslinked RNCs Figure 5b, righthandside plot)**

	PIR 30	PIR 40	PIR 50	PIR 60	PIR 70	PIR 80	PIR 91
PIR 30	-----						
PIR 40	0.86	-----					
PIR 50	0.36	0.15	-----				
PIR 60	0.15	0.03	0.35	-----			
PIR 70	0.03	0.00	0.01	0.10	-----		
PIR 80	0.00	0.03	0.00	0.00	0.00	-----	
PIR 91	0.00	0.00	0.00	0.00	0.00	0.00	-----

same / different

Fig. S5. P values for the two-tailed Student's t-test resulting from the comparison of data in the (a), lefthandside and (a), righthandside plots of Fig. 5b. Values enclosed in green boxes denote statistically different crosslinking fractions within ≥95% confidence.

Fig. S6

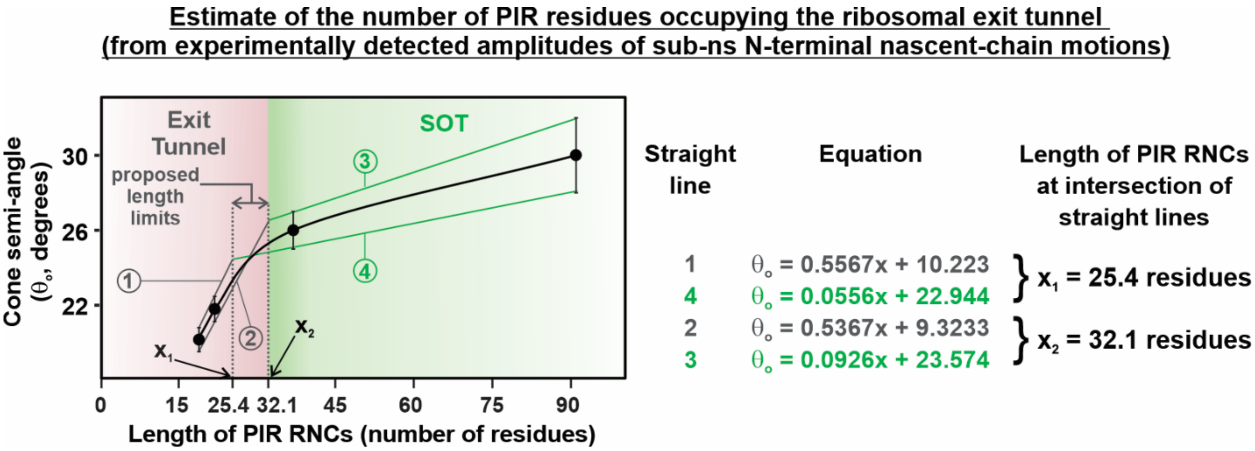

Fig. S6. Analysis of previously reported[20] amplitudes of N-terminal sub-ns motions of nascent PIR_{19}, PIR_{22}, PIR_{37} and PIR_{91} chains. The four straight lines, denoted as 1-4, describe nascent-chain cone semi-angles across different regions of the ribosomal exit tunnel or the surface outside the tunnel (SOT, according to Model I of Fig. 5f) upon taking into account experimental standard errors (S.E.). Intersections between lines 1 and 4 (X_1) and between lines 2 and 3 (X_2) provide estimates of the lower- (X_1) and upper- (X_2) limit values for the number of PIR-RNC residues within the ribosomal exit-tunnel core. X_1 and X_2 were found to be 25.4 and 32.1, respectively.

Fig. S7

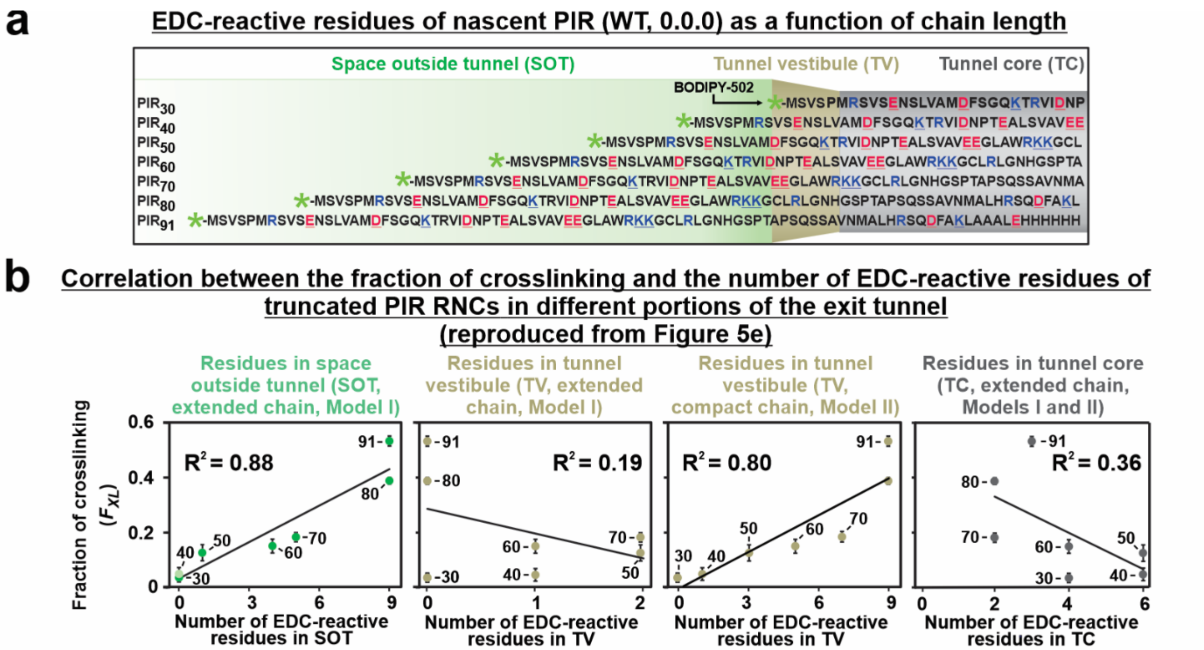

c Number of EDC-reactive residues of PIR RNCs in different regions of the ribosome

space outside tunnel (SOT) tunnel vestibule (TV) tunnel core (TC)

Length of PIR RNC	SOT, extended chain Model I	TV, extended chain Model I	TV, compact chain Model II	TC, extended chain Models I and II
30	0	0	0	4
40	0	1	1	6
50	1	2	3	6
60	4	1	5	4
70	5	2	7	2
80	9	0	9	2
91	9	0	9	3

d Linear regression parameters of data in panel b

space outside tunnel (SOT) tunnel vestibule (TV) tunnel core (TC)

Regions	R^2	slope	intercept
SOT, extended chain (Model I)	0.88	0.04 ± 0.01	0.02 ± 0.04
TV, extended chain (Model I)	0.19	-0.09 ± 0.08	0.30 ± 0.09
TV, compact (Model II)	0.80	0.05 ± 0.01	-0.01 ± 0.06
TC, extended / compact chain (Models I and II)	0.36	-0.08 ± 0.04	0.50 ± 0.15

Fig. S7. Correlation analysis assessing whether the fraction of crosslinking correlates with the exposure of EDC-reactive residues to the space outside the ribosomal tunnel (SOT, Model I of Fig. 5f) or with the space within the ribosomal vestibule (TV, Model II of Fig. 5f). (a), Schematic illustration of the amino acid sequences of PIR_{30} WT to PIR_{91} WT RNCs. Residues with acidic and basic side chains are shown in red and blue, respectively. EDC-reactive residues are underlined. The space outside the tunnel (SOT), within the tunnel vestibule (TV) and within the tunnel core (TC) according to Model I of Fig. 5f are shown in light green, tan, and gray, respectively. **(b),** Assessment of linear dependence between the fraction of crosslinked RNCs and the number of EDC-reactive residues in the SOT (**left**), TV **(middle)**, and TC **(right)**, respectively, for either extended (Model I of Fig. 5f) or compact ribosome-interacting (Model II of Fig. 5f) nascent chains. **(c),** Number of EDC-reactive residues of PIR RNCs in different regions of the ribosome for either extended (Model I of Fig. 5f) or compact ribosome-interacting (Model II of Fig. 5f) nascent chains. **(d),** Linear regression parameters for the data in panel b.

Fig. S8

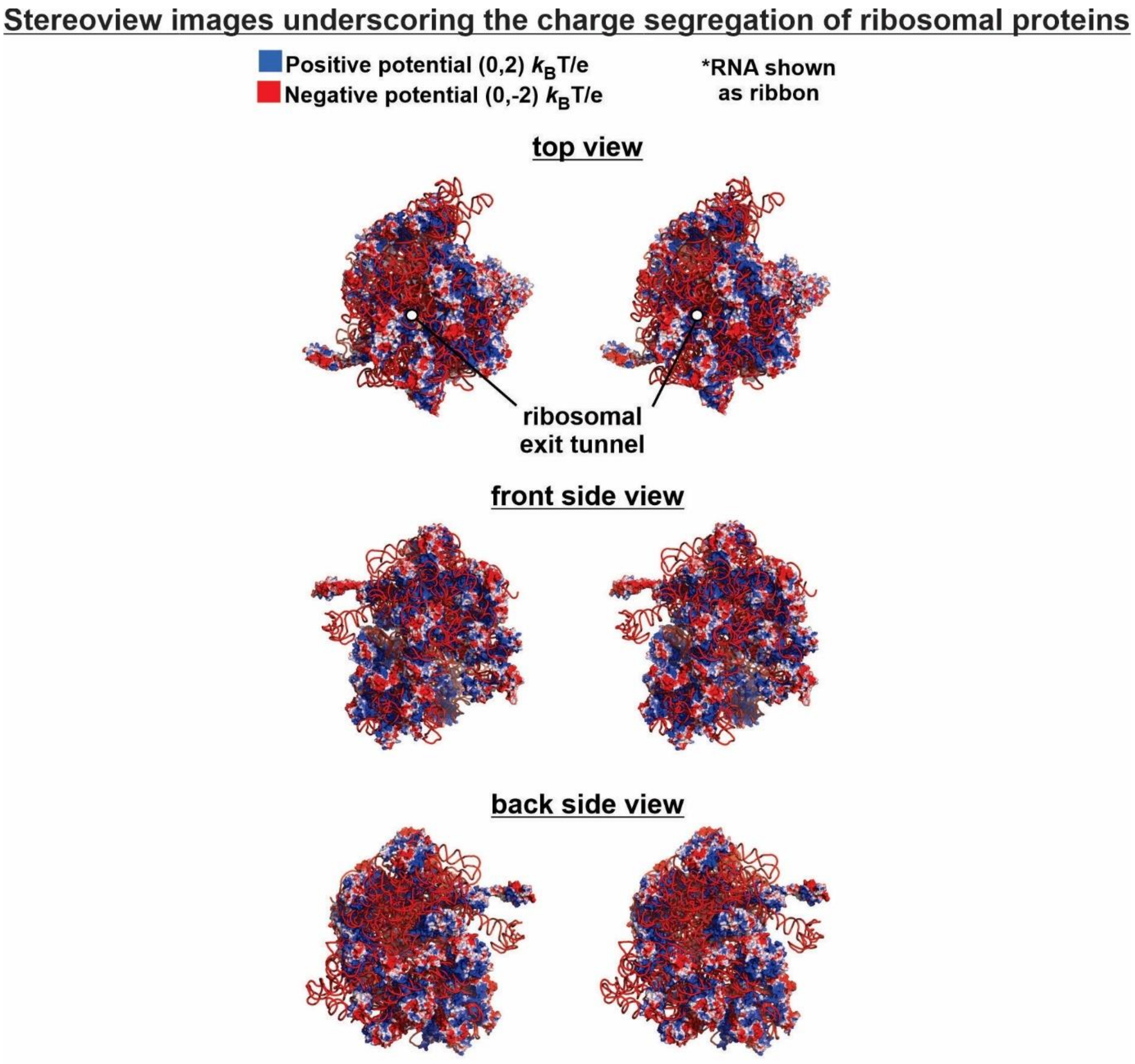

Fig. S8. Electrostatic surface-potential maps of proteins belonging to the *E. coli* 70S ribosome. Ribosomal RNA (16S, 23S, and 5S) and ribosomal proteins are shown as red ribbons and solid surfaces, respectively. Ribosomal proteins show a clear charge-segregation pattern according to which regions with negative electrostatic potential (- 5 to 0 k_BT/*e*) face outward (i.e., towards the solvent), and regions with positive electrostatic potential (0 to + 5 k_BT/*e*) face the ribosomal core. See Movie S2 for a more interactive rendering. Electrostatic surface potentials were computed according to Fedyukina *et al.*[21] via APBS (150 mM ionic strength, solute dielectric = 2.0, and solvent dielectric = 78.0) starting from the three-dimensional structure of the *E. coli* ribosome (PDB ID 4YBB). Regions with positive (0 to +5 k_BT/*e*) and negative (-5 to 0 k_BT/*e*) surface potential are shown in blue and red, respectively. k_BT denotes an energy of 4.11x10-21 J at room temperature, where k_B is the Boltzmann constant (in J/K) and T is the temperature (in K), and *e* denotes the electric charge (in Coulomb).

Fig. S9

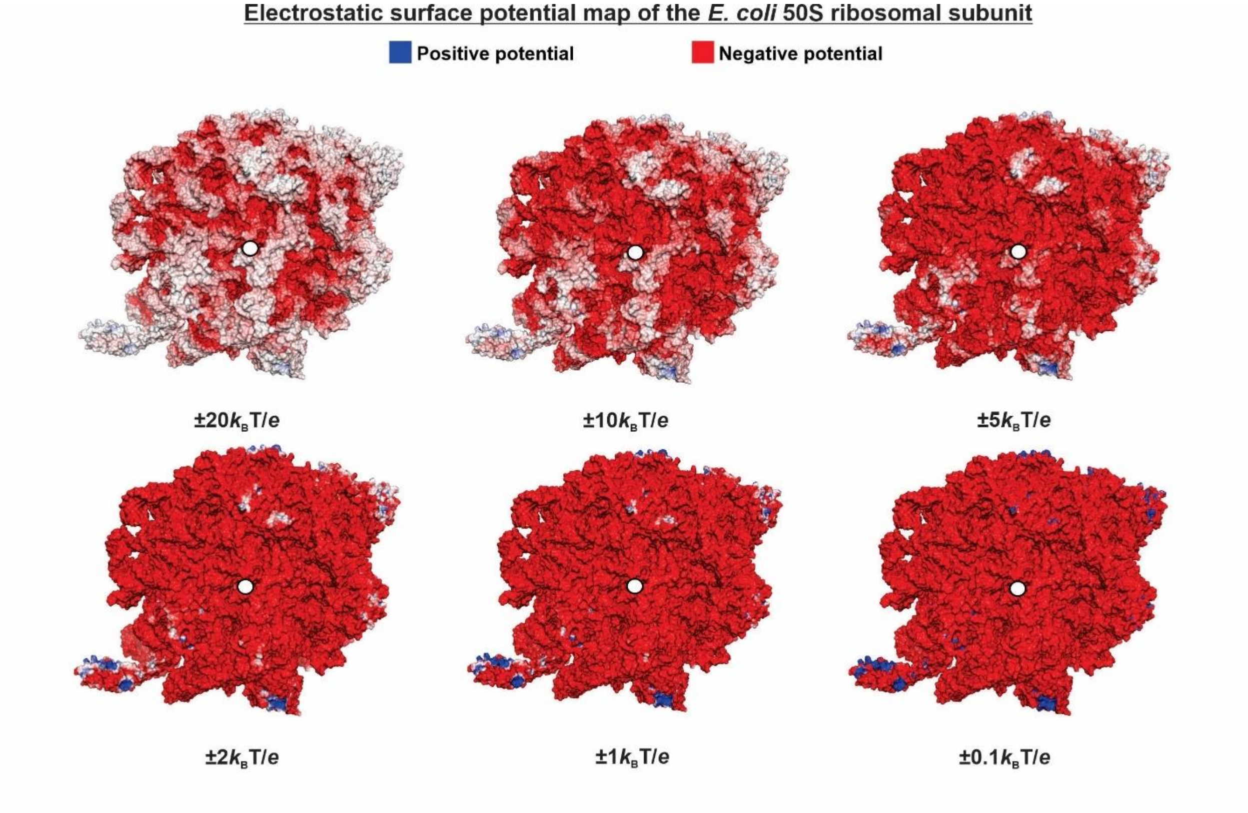

Fig. S9. Electrostatic surface potential maps of 50S *E. coli* ribosomal subunit color-coded according to different k_BT/*e* ranges. Full electrostatic surface potential maps (accounting for both ribosomal RNA and ribosomal proteins) are shown. k_BT denotes an energy unit of $4.11x10^{-21}$ J at room temperature, where k_B is the Boltzmann constant and T is temperature, and *e* denotes the electric charge in Coulombs. Positive and negative potentials are shown in blue and red, respectively, while regions with k_BT/*e* = 0 are shown in white. Electrostatic surface potentials were computed according to Fedyukina *et al.*[21] via APBS (150 mM ionic strength, solute dielectric = 2.0, solvent dielectric = 78.0) starting from the three-dimensional structure of the *E. coli* ribosome (PDB ID 4YBB).

Fig. S10

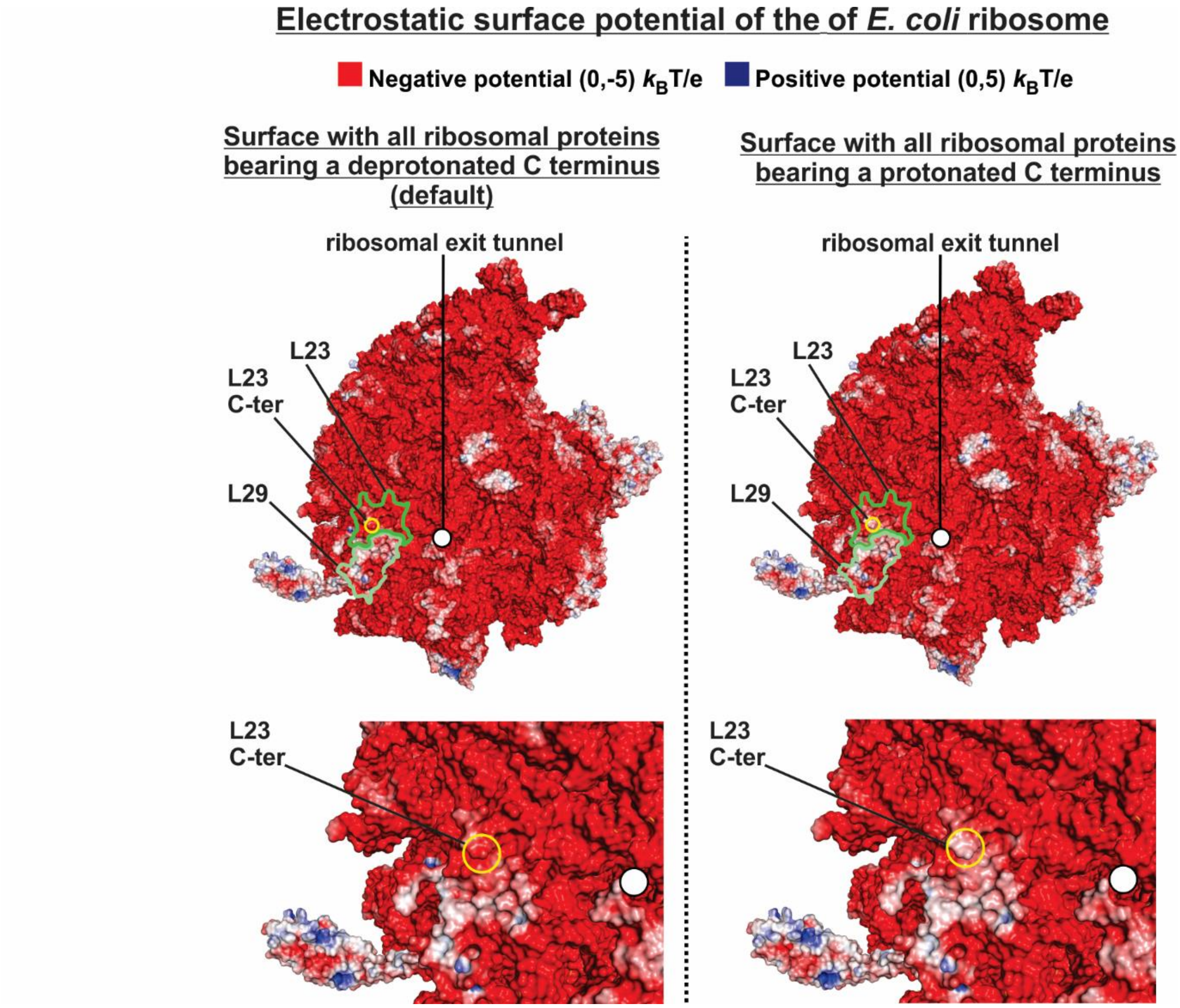

Fig. S10. Electrostatic surface potentials of *E. coli* ribosomes with deprotonated (lefthandside) and protonated (righthandside) C termini of all ribosomal proteins. Electrostatic surface potentials were computed as in Fig. S9. Abbreviations are as in Fig. S9. Regions with positive (0 to +5 $k_B T/e$) and negative electrostatic surface potential (-5 to 0 $k_B T/e$) are shown in blue and red, respectively. Uncharged regions ($k_B T/e$ = 0) are white. The surfaces located on the left and right portions of the figure correspond to ribosomes with all ribosomal proteins bearing a deprotonated and protonated C terminus, respectively. The images on the lower portion of the figures show close-up views highlighting the C terminus of the L23 ribosomal protein.

Fig. S11

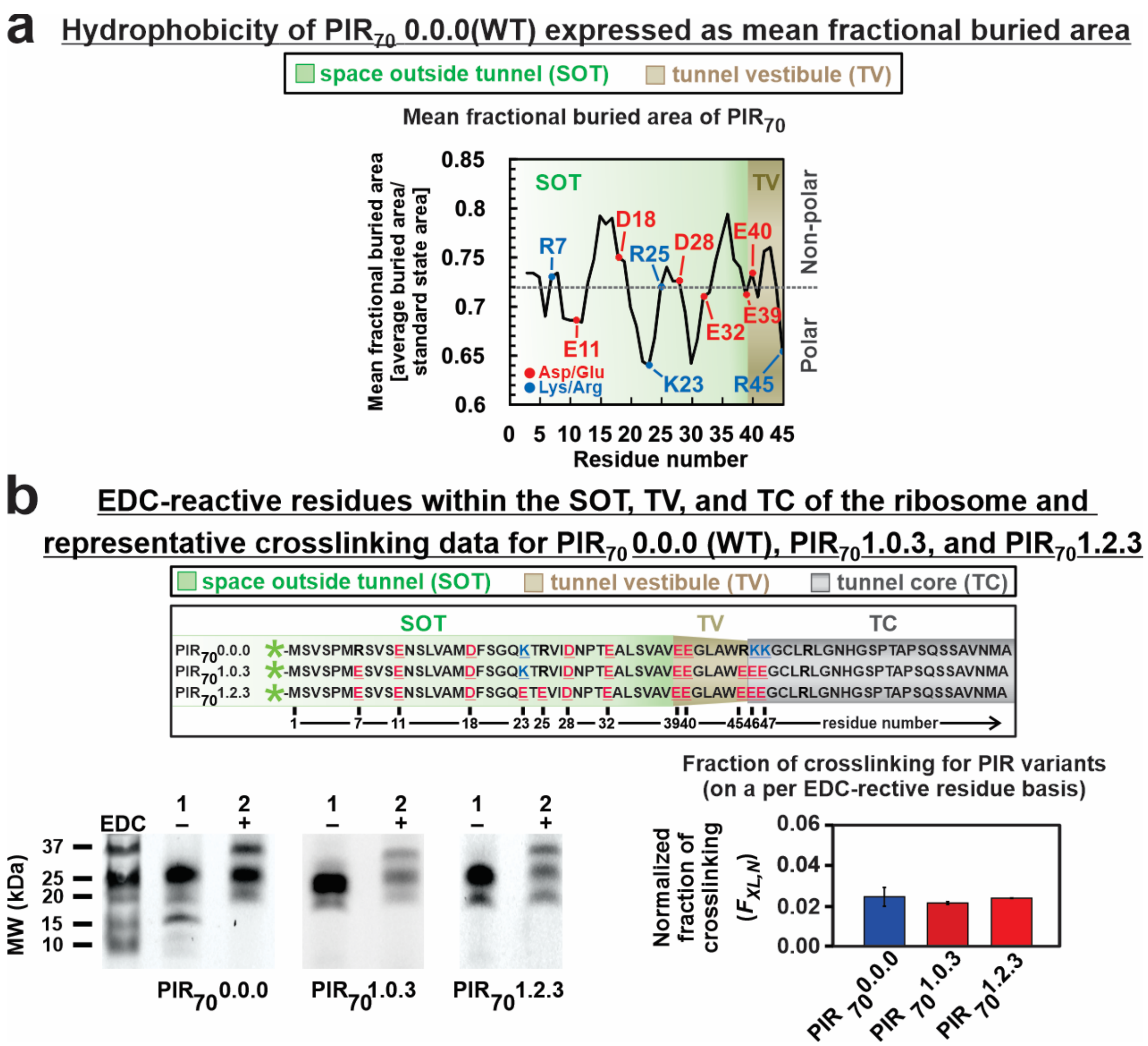

Fig. S11. Crosslinking data for WT and variant incomplete PIR nascent chains of bearing 70 residues (PIR_{70}). (a), Hydrophobicity of solvent-exposed portion of PIR_{70} according to Model I of Fig. 5f. Mean buried area scores were computed according to Rose *et al*.[22] with a 5-residue window. **(b),** Top: Schematic illustration of amino acid sequence of PIR_{70} RNCs, including WT 0.0.0 PIR_{70}, and the 1.0.3 PIR_{70}, and 1.2.3 PIR_{70} variants. The background coloring of the surface outside the tunnel (SOT), tunnel vestibule (TV) and tunnel core (TC) assumes a partitioning consistent with Model I of Fig. 5f. Residues D/E and K/R are shown in red and blue. EDC-reactive residues are underlined. Bottom: Representative gel and normalized fraction of crosslinking for WT 0.0.0 PIR_{70}, and 1.0.3 PIR_{70} and 1.2.3 PIR_{70} variants. Error bars in the bar graph denote ± SE for n =2.

Supplementary References

1. Cruz-Vera, L. R., Rajagopal, S., Squires, C. & Yanofsky, C. Features of ribosome-peptidyl-tRNA interactions essential for tryptophan induction of tna operon expression. *Mol. Cell* **19**, 333-343 (2005).
2. Seidelt, B. et al. Structural insight into nascent polypeptide chain-mediated translational stalling. *Science* **326**, 1412-1415 (2009).
3. Houben, E. N., Zarivach, R., Oudega, B. & Luirink, J. Early encounters of a nascent membrane protein: specificity and timing of contacts inside and outside the ribosome. *J. Cell Biol.* **170**, 27-35 (2005).
4. Bhushan, S. et al. SecM-stalled ribosomes adopt an altered geometry at the peptidyl transferase center. *PLoS Biol.* **9**, e1000581 (2011).
5. Nakatogawa, H. & Ito, K. The ribosomal exit tunnel functions as a discriminating gate. *Cell* **108**, 629-636 (2002).
6. Bhushan, S. et al. Structural basis for translational stalling by human cytomegalovirus and fungal arginine attenuator peptide. *Mol. Cell* **40**, 138-146 (2010).
7. Zhang, Y., Wolfle, T. & Rospert, S. Interaction of nascent chains with the ribosomal tunnel proteins Rpl4, Rpl17, and Rpl39 of Saccharomyces cerevisiae. *J. Biol. Chem.* **288**, 33697-33707 (2013).
8. Woolhead, C. A., McCormick, P. J. & Johnson, A. E. Nascent Membrane and Secretory Proteins Differ in FRET-Detected Folding Far inside the Ribosome and in Their Exposure to Ribosomal Proteins. *Cell* **116**, 725-736 (2004).
9. Ullers, R. S. et al. Interplay of signal recognition particle and trigger factor at L23 near the nascent chain exit site on the Escherichia coli ribosome. *J. Cell Biol.* **161**, 679-684 (2003).
10. Eisner, G. et al. Alternate Recruitment of Signal Recognition Particle and Trigger Factor to the Signal Sequence of a Growing Nascent Polypeptide. *J. Biol. Chem.* **281**, 7172-7179 (2006).
11. Peterson, J. H., Woolhead, C. A. & Bernstein, H. D. The conformation of a nascent polypeptide inside the ribosome tunnel affects protein targeting and protein folding. *Mol. Microbiol.* **78**, 203-217 (2010).
12. Wang, S. et al. The molecular mechanism of cotranslational membrane protein recognition and targeting by SecA. *Nat. Struct. Mol. Biol.* **26**, 919-+ (2019).
13. Arnold, R. J. & Reilly, J. P. Observation of *Escherichia coli* ribosomal proteins and their posttranslational modifications by mass spectrometry. *Anal. Biochem.* **269**, 105-112 (1999).
14. Hoffmann, A., Bukau, B. & Kramer, G. Structure and function of the molecular chaperone Trigger Factor. *Biochimica Et Biophysica Acta-Molecular Cell Research* **1803**, 650-661 (2010).
15. Lill, R., Crooke, E., Guthrie, B. & Wickner, W. The trigger factor cycle includes ribosomes, Presecretory proteins, and the plasma membrane. *Cell* **54**, 1013-1018 (1988).
16. Hesterkamp, T. & Bukau, B. Role of the DnaK and HscA homologs of Hsp70 chaperones in protein folding in E-coli. *EMBO J.* **17**, 4818-4828 (1998).
17. Ellis, R. J. & Hartl, F. U. Protein folding in the cell: Competing models of chaperonin function. *FASEB J.* **10**, 20-26 (1996).
18. Rudiger, S., Germeroth, L., SchneiderMergener, J. & Bukau, B. Substrate specificity of the DnaK chaperone determined by screening cellulose-bound peptide libraries. *EMBO J.* **16**, 1501-1507 (1997).
19. Dalphin, M. D., Stangl, A. J., Liu, Y. & Cavagnero, S. KLR-70: A Novel Cationic Inhibitor of the Bacterial Hsp70 Chaperone. *Biochemistry* **59**, 1946-1960 (2020).

20. Ellis, J. P., Culviner, P. H. & Cavagnero, S. Confined dynamics of a ribosome-bound nascent globin: Cone angle analysis of fluorescence depolarization decays in the presence of two local motions. *Protein Sci.* **18**, 2003-2015 (2009).
21. Fedyukina, D. V., Jennaro, T. S. & Cavagnero, S. Charge segregation and low hydrophobicity are key features of ribosomal proteins from different organisms. *J. Biol. Chem.* **289**, 6740-6750 (2014).
22. Rose, G. D. et al. Hydrophobicity of amino acid residues in globular proteins. *Science* **229**, 834-838 (1985).
