# Supplementary material for: An intrinsically disordered nascent protein interacts with specific regions of the ribosomal surface near the exit tunnel
Source: Commun Biol. 2021 Oct 29;4:1236. doi: 10.1038/s42003-021-02752-4 (PMC8556260; doi:10.1038/s42003-021-02752-4)
Supplement: Supplementary file 3 — Description of Additional Supplementary Files [file 42003_2021_2752_MOESM3_ESM.pdf]

Description of Additional Supplementary Files

File name: Supplementary Movie S1.

Description: This movie shows a close-up view of the L23, L24 and L29 ribosomal proteins within the E. coli 50S ribosomal subunit (PDB ID 4YBB). All of these proteins lie near the end of the ribosomal exit tunnel. Ribosomal RNA is shown as red ribbons. Nonpolar amino acids (Phe, Ile, Leu, Met, Ala, Val, Gly, Cys, Pro, Trp) are shown in tan and charged residues are shown in blue (Lys, Arg) or red (Asp, Glu).

File name: Supplementary Movie S2.

Description: Movie highlighting the charge segregation of the ribosomal proteins within the E. coli ribosome. Ribosomal RNA (16S, 23S, and 5S) and ribosomal proteins are shown as red ribbons and solid surfaces, respectively. In analogy with the images in Figure S8, ribosomal proteins show a clear charge-segregation pattern according to which regions with negative electrostatic potential (- 2 to 0 kBT/e) face outward (i.e., towards the solvent), and regions with positive electrostatic potential (0 to + 2 kBT/e) face the ribosomal core. Electrostatic surface potentials were computed according to Fedyukina et al.21 via APBS (150 mM ionic strength, solute dielectric = 2.0, and solvent dielectric = 78.0) starting from the threedimensional structure of the E. coli ribosome (PDB ID 4YBB). Regions with positive (0 to +5 kBT/e) and negative (-5 to 0 kBT/e) surface potential are shown in blue and red, respectively. kBT denotes an energy of 4.11x10-21 J at room temperature, where kB is the Boltzmann constant (in J/K) and T is the temperature (in K), and e denotes the electric charge (in Coulomb).

File name: Supplementary Data 1.

Description: Source data for the graphs and charts in the main figures.
