# Supplementary material for: An intrinsically disordered nascent protein interacts with specific regions of the ribosomal surface near the exit tunnel
Source: Commun Biol. 2021 Oct 29;4:1236. doi: 10.1038/s42003-021-02752-4 (PMC8556260; doi:10.1038/s42003-021-02752-4)
Supplement: Supplementary file 7 — Reporting Summary [file 42003_2021_2752_MOESM7_ESM.pdf]

nature research

Corresponding author(s): Cavagnero, S.

Last updated by author(s): 8/11/2021

Reporting Summary

Nature Research wishes to improve the reproducibility of the work that we publish. This form provides structure for consistency and transparency in reporting. For further information on Nature Research policies, see our Editorial Policies and the Editorial Policy Checklist.

Statistics

For all statistical analyses, confirm that the following items are present in the figure legend, table legend, main text, or Methods section.

n/a	Confirmed	
☐	☒	The exact sample size (*n*) for each experimental group/condition, given as a discrete number and unit of measurement
☐	☒	A statement on whether measurements were taken from distinct samples or whether the same sample was measured repeatedly
☐	☒	The statistical test(s) used AND whether they are one- or two-sided<br>*Only common tests should be described solely by name; describe more complex techniques in the Methods section.*
☒	☐	A description of all covariates tested
☒	☐	A description of any assumptions or corrections, such as tests of normality and adjustment for multiple comparisons
☐	☒	A full description of the statistical parameters including central tendency (e.g. means) or other basic estimates (e.g. regression coefficient) AND variation (e.g. standard deviation) or associated estimates of uncertainty (e.g. confidence intervals)
☐	☒	For null hypothesis testing, the test statistic (e.g. *F*, *t*, *r*) with confidence intervals, effect sizes, degrees of freedom and *P* value noted<br>*Give P values as exact values whenever suitable.*
☒	☐	For Bayesian analysis, information on the choice of priors and Markov chain Monte Carlo settings
☒	☐	For hierarchical and complex designs, identification of the appropriate level for tests and full reporting of outcomes
☒	☐	Estimates of effect sizes (e.g. Cohen's *d*, Pearson's *r*), indicating how they were calculated

Our web collection on statistics for biologists contains articles on many of the points above.

Software and code

Policy information about availability of computer code

Data collection	No particular software was used to collect the data
Data analysis	Excel (Microsoft, 2016), Coreldraw 2018, CorelPhotoPaint 2018, Kaleidagraph 3.5, COPASI 4.22, Pymol2.3.3, ImageJ 1.5

For manuscripts utilizing custom algorithms or software that are central to the research but not yet described in published literature, software must be made available to editors and reviewers. We strongly encourage code deposition in a community repository (e.g. GitHub). See the Nature Research guidelines for submitting code & software for further information.

Data

Policy information about availability of data

All manuscripts must include a data availability statement. This statement should provide the following information, where applicable:

- Accession codes, unique identifiers, or web links for publicly available datasets
- A list of figures that have associated raw data
- A description of any restrictions on data availability

The data that support the findings of this study are available from the corresponding author upon reasonable request.

Field-specific reporting

Please select the one below that is the best fit for your research. If you are not sure, read the appropriate sections before making your selection.

☒ Life sciences ☐ Behavioural & social sciences ☐ Ecological, evolutionary & environmental sciences

For a reference copy of the document with all sections, see nature.com/documents/nr-reporting-summary-flat.pdf

Life sciences study design

All studies must disclose on these points even when the disclosure is negative.

Sample size	Sample sizes were determined based on previous publications.
Data exclusions	No data were excluded
Replication	All attempts at replication were successful
Randomization	Randomization is not relevant to this study
Blinding	Blinding is not relevant to this study

Reporting for specific materials, systems and methods

We require information from authors about some types of materials, experimental systems and methods used in many studies. Here, indicate whether each material, system or method listed is relevant to your study. If you are not sure if a list item applies to your research, read the appropriate section before selecting a response.

Materials & experimental systems

n/a	Involved in the study	
☐	☒	Antibodies
☒	☐	Eukaryotic cell lines
☒	☐	Palaeontology and archaeology
☒	☐	Animals and other organisms
☒	☐	Human research participants
☒	☐	Clinical data
☒	☐	Dual use research of concern

Methods

n/a	Involved in the study	
☒	☐	ChIP-seq
☒	☐	Flow cytometry
☒	☐	MRI-based neuroimaging

Antibodies

Antibodies used

In this study, we employed antibodies against the L17, L18-L22, L23, L24, L29, L32 E. coli ribosomal proteins. The primary antibodies against these E. coli ribosomal proteins were donated by the scientists below.

Anti-uL23 antibodies:
Prof. Shu-ou Shan (California Institute of Technology) kindly donated rabbit anti-uL23 antibodies. These antibodyies had been custom-ordered from GenScript, using the CGKVKRHGQRIGRRS peptide as epitope. The antibodies were employed in previously published research (see Validation section below).

Anti-uL17, uL18-uL22, uL24, uL29, uL32 antibodies:
Prof. Bryan W. Davies (University of Texas at Austin) kindly donated rabbit anti-uL17, uL18/L22, uL24, uL29 and uL32 antibodies. These antibodies had been previously generated within Prof. Masayasu Nomura's research group. The purified individual ribosomal proteins L17, L23, L24, L29, and L32 were injected into rabbits as antigens. For proteins L18 and L22, individual protein purification was not possible, thus a mixture of them was used, leading to a combined response to both L18 and L22. Given that Prof. Nomura died in 2011, Melanie Oakes (University of California-Irvine), who took over Dr. Masayasu Nomura's research after his death, kindly provided us with antibody preparation protocols that include rabbit number, bleeding, MW, chromatography details including a summary o HPLC procedures. These antibodies were employed in previously published research (see Validation section below).

Please note that specific information regarding prior publications employing the above antibodies will be added to our manuscript after the initial review process has been completed.

Validation

Anti-uL23 antibodies:
Anti-uL23 antibodies were obtained from Shu-ou Shan, as listed in the previous section. An initial validation criterion is the fact that these antibodies were raised by GenScript, using the CGKVKRHGQRIGRRS peptide as epitope. This peptide is a fragment of the E. coli L23 protein. Therefore the antibodies were raised to specifically react to the presence of L23. In addition, a recent article from the Shu-ou Shan group, employing these same antibodies, showed that in E. coli cell-free extracts these antibodies exhibits affinity for a

proteins with the same molecular weight as L23. In addition, these antibodies displayed affinity for three other proteins with higher molecular weight (1). In our own Western blots (manuscript Figure 3), however, we did not observe any of this undesired cross-reactivity (see bands 1 and 2 in Western bots of Figure 3). This is probably because in our work we used purified ribosome-bound nascent chains (RNCs, sucrose cushion-purified and resuspended in buffer), which do not contain any of the undesired cross-reacting proteins.

Anti-uL17, -uL18/L22, -uL24, -uL29, and -uL32 antibodies:
First of all, the identity and reactivity of these antibodies is consistent with the fact that they were raised in rabbits, against purified individual ribosomal proteins L17, L23, L18+L22, L24, L29, and L32. The specificity of each antiserum was assessed via Ouchterlony immunodiffusion (2) against all ribosomal proteins (3). The authors observed a response only against the protein(s) used as antigen for raising the antiserum. Finally, the same antibodies that we used in our research were used in previous published studies (3 and 4).

References:
(1) Wang S, Jomaa A, Jaskolowski M, Yang CI, Ban N, Shan SO. The molecular mechanism of cotranslational membrane protein recognition and targeting by SecA. Nat Struct Mol Biol. 2019;26(10):919-929. doi:10.1038/s41594-019-0297-8
(2) Bailey G.S. (1996) Ouchterlony Double Immunodiffusion. In: Walker J.M. (eds) The Protein Protocols Handbook. Springer Protocols Handbooks. Humana Press
(3) Lindahl L, Post L, Zengel J, Gilbert SF, Strycharz WA, Nomura M. Mapping of ribosomal protein genes by in vitro protein synthesis using DNA fragments of lambdafus3 transducing phage DNA as templates. J Biol Chem. 1977;252(20):7365-7383.
(4) Fahnestock SR, Strycharz WA, Marquis DM. Immunochemical evidence of homologies among 50 S ribosomal proteins of Bacillus stearothermophilus and Escherichia coli. J Biol Chem. 1981;256(19):10111-10116.
